# Perceptions of measles, pneumonia, and meningitis vaccines among caregivers in Shanghai, China, and the health belief model: a cross-sectional study

**DOI:** 10.1186/s12887-017-0900-2

**Published:** 2017-06-12

**Authors:** Abram L. Wagner, Matthew L. Boulton, Xiaodong Sun, Bhramar Mukherjee, Zhuoying Huang, Irene A. Harmsen, Jia Ren, Brian J. Zikmund-Fisher

**Affiliations:** 1 Department of Epidemiology, University of Michigan, 1415 Washington Heights, Ann Arbor, MI 48109 USA; 2 Department of Immunization Programs, Shanghai Centers for Disease Control and Prevention, 1380 Zhongshan West Road, Shanghai, 200336 China; 3 Department of Biostatistics, University of Michigan, Ann Arbor, 1415 Washington Heights, Ann Arbor, MI 48109 USA; 4 Department of Epidemiology and Health Promotion, Public Health Service of Amsterdam, Amsterdam, the Netherlands; 5 Department of Health Behavior and Health Education, University of Michigan, Ann Arbor, 1415 Washington Heights, Ann Arbor, MI 48109 USA

**Keywords:** Health belief model, Immunization coverage, China, Measles, Pneumococcus

## Abstract

**Background:**

In China, the measles vaccine is offered for free whereas the pneumococcal vaccine is a for-fee vaccine. This difference has the potential to influence how caregivers evaluate whether a vaccine is important or necessary for their child, but it is unclear if models of health behavior, such as the Health Belief Model, reveal the same associations for different diseases. This study compares caregiver perceptions of different diseases (measles, pneumonia and meningitis); and characterizes associations between Health Belief Model constructs and both pneumococcal vaccine uptake and perceived vaccine necessity for pneumonia, measles, and meningitis.

**Methods:**

Caregivers of infants and young children between 8 months and 7 years of age from Shanghai (*n* = 619) completed a written survey on their perceptions of measles, pneumonia, and meningitis. We used logistic regression models to assess predictors of pneumococcal vaccine uptake and vaccine necessity.

**Results:**

Only 25.2% of children had received a pneumococcal vaccine, although most caregivers believed that pneumonia (80.8%) and meningitis (92.4%), as well as measles (93.2%), vaccines were serious enough to warrant a vaccine. Perceived safety was strongly associated with both pneumococcal vaccine uptake and perceived vaccine necessity, and non-locals had 1.70 times higher odds of pneumonia vaccine necessity than non-locals (95% CI: 1.01, 2.88).

**Conclusions:**

Most factors had a similar relationship with vaccine necessity, regardless of disease, indicating a common mechanism for how Chinese caregivers decided which vaccines are necessary. Because more caregivers believed meningitis needed a vaccine than pneumonia, health care workers should emphasize pneumococcal vaccination’s ability to protect against meningitis.

**Electronic supplementary material:**

The online version of this article (doi:10.1186/s12887-017-0900-2) contains supplementary material, which is available to authorized users.

## Background

The World Health Organization promotes the global adoption of new vaccines through its Expanded Program on Immunization (EPI) [1, 2], although individual countries decide which vaccines to include based on local epidemiological, financial, and other considerations. The EPI in China started in 1978 and included the tuberculosis, polio, measles, and diphtheria-tetanus-pertussis (DTP) vaccines. Since then, it has expanded to include hepatitis A and B, meningococcal, Japanese encephalitis, rubella, and mumps vaccines [1]. All EPI vaccines in China are free and mandatory for school entry.

Immunization clinics in China also offer non-EPI vaccines to children for a fee (and not covered by insurance programs), including influenza, varicella, *Haemophilus influenzae* type b (Hib), rotavirus, and pneumococcal vaccines, among others. The pneumococcal vaccine, in particular, is a prime candidate for inclusion on the EPI schedule given the substantial burden of pneumococcal disease in China [3]. It has been introduced in many low-income countries with support from Gavi, the Vaccine Alliance [4], and it could prevent some of the 261,000 cases and 11,000 deaths due to pneumococcal pneumonia and meningitis in Chinese children under 5 years of age annually [5]. These figures are greater than, for example, the number of measles cases in China, which have fluctuated between 2005 and 2013 from a high of 123,136 in 2005 to a low of 6183 in 2012 [6].

Non-EPI vaccines have lower childhood coverage than EPI vaccines in China; for example, coverage of the 7-valent pneumococcal conjugate vaccine (PCV7) is 10.1% and coverage of the 23-valent pneumococcal polysaccharide vaccine (PPSV23) is 29.8% in Shanghai, which are both non-EPI vaccines, compared to >97% for DTP, an EPI vaccine [7, 8]. This disparity arises in part because of their expense [9]; for example, PCV7 costs approximately $135 per dose and PPSV23 is approximately $24 per dose. A study from 2013 in Jiangsu, Hubei, and Gansu provinces, found that the median amount that caregivers were willing to pay for the pneumococcal vaccine was between 150 and 200 RMB ($20–$30) [10]. Given the current lack of government funding for pneumococcal vaccination, understanding Chinese caregivers’ perceptions about this non-EPI vaccine and the diseases it prevents is key to developing effective interventions to increase vaccine uptake. And, if pneumococcal vaccine is added to the EPI schedule, understanding these perceptions will be important for developing effective programs to increase people’s acceptance of the vaccine.

Vaccine decision-making can be explained by health behavior models like the Health Belief Model (HBM) [11], which conceives of vaccination behaviors as an output of an individual’s perceptions of both a disease and its related vaccine [12]. These constructs specifically include people’s perceived susceptibility or vulnerability to the disease (i.e., the subjective perception of the risk associated with getting the disease), their understanding of disease severity (which could include medical consequences like disability and death or social consequences such as limited social interactions), a sense of the potential benefits of vaccination (e.g., effectiveness of vaccines), and anticipated barriers to vaccination (financial and temporal cost, side effects, unpleasant/painful injection) [12, 13]. Vaccine decision-making can also be influenced by demographic characteristics, such as residency and urbanicity. Non-locals, or migrants from rural areas to urban cities [14], have less access to governmental entitlement programs than locals [14, 15]. but still receive EPI vaccines for free; and urban districts represent historical business areas, whereas suburban districts are more industrial and have less access to public services [16, 17].

Although previous studies in China have shown the usefulness of an HBM framework for understanding perceived dysentery vaccine need [18], influenza vaccination intent [19], and influenza vaccine uptake among healthcare workers [20], no previous study in China has contrasted perceptions between EPI and non-EPI vaccines among caregivers using the HBM. It may be that people think differently about vaccines, such as the measles and pneumococcal vaccines, which have divergent payment mechanisms, which vary by length of time on the market, and for which people plausibly have different levels of personal experience. In this study, we compare perceptions of measles, pneumonia, and meningitis vaccines among caregivers in Shanghai; we characterize the associations between HBM constructs and pneumococcal vaccine uptake; and we contrast the associations between HBM constructs and perceived vaccine necessity of measles, pneumonia, and meningitis.

## Methods

### Study population

In this cross-sectional study which was completed during May and June of 2014, we invited caregivers (i.e., parents or grandparents) of young children at immunization clinics in Shanghai to participate in a survey that focused on their perceptions of vaccines for measles, pneumonia, and meningitis. We selected caregivers into the study through a two-stage, stratified, cluster sampling. The sample size was based on another aim of the project (to discriminate between measles vaccination timeliness of 81% in non-locals and 91% in locals), which required a simple random size of 208 per group or 416 total. Using another dataset on measles vaccination timeliness [21], we estimated an intracluster correlation coefficient of 0.024, and with a desired sample of 20 per cluster, we estimated a design effect of 1.456 for an effective sample size of 606. Clusters in this sample refer to townships, administrative regions in China which have an immunization clinic. There were 230 townships in Shanghai listed in the Census; we excluded 21 from Chongming county—islands off the coast of Shanghai which are distant from the other counties in the city, for a total of 209 townships in our selection. Townships were selected by a probability proportionate to size (PPS) systematic selection procedure with population of children 0 to 14 years of age from the China 2010 Census as the population size.

Within each township immunization clinic (where individuals obtain EPI and non-EPI vaccines), we selected a convenience sample, in person, of at least 20 caregivers who accompanied their child for a vaccination visit. The sole eligibility criterion was that the child was between 8 months and 7 years of age, which made them eligible for receipt of the measles and pneumococcal vaccines. We attempted to sample an equal number of locals and non-locals at each clinic because of hypothesized differences in experience with disease between the two groups. All potential participants gave informed consent prior to completing the paper survey at the immunization clinic. The survey was in Chinese and took approximately 20 min to complete, and participants were given an incentive of 30 renminbi ($5). An English version of the questionnaire is available in Additional file 1. The analysis included sampling weights derived from the township selection probability and the proportion of non-locals and locals in the township so that our study population resembled the population structure of locals and non-locals in Shanghai.

### Questionnaire

The questionnaire collected information on caregiver perceptions of pediatric vaccines, in general, and measles and pneumococcal vaccines, more specifically. The questions were informed by previous literature on beliefs and perceptions of vaccine-preventable diseases [22–27], in addition to a qualitative, pilot research project undertaken by the lead author on 23 parents and grandparents at immunization clinics in Tianjin, China, during the summer of 2013 [28]. Prior to data collection, the questionnaire underwent pre-testing with 10 native Chinese speakers in the United States and 9 parents living in China. The questionnaire was also piloted in one township clinic in Shanghai. Questions were revised based on feedback in these pre-test settings. For a portion of the questionnaire, the same questions were asked about all three diseases (hereafter indicated as [disease type]): measles, pneumonia, or meningitis.

### Outcome variables

The first outcome considered was pneumococcal vaccine uptake, which was administration of at least one dose of pneumococcal conjugate vaccine or pneumococcal polysaccharide vaccine, as documented in the child’s vaccination booklet. Because coverage of measles vaccine, which is part of the EPI, approaches 100% in China, we chose another outcome to allow us to compare how people make decisions about both measles and pneumococcal vaccines. This outcome, “vaccine necessity,” was the response to the question “Do you think that [disease type] is a serious enough disease to warrant a vaccine?”

### Predictor variables

Local or non-local status was based on a previously completed field in the child’s vaccination booklet. Urbanicity was based on the location of the clinic: the urban districts include Huangpu, Xuhui, Changning, Jing’an, Putuo, Zhabei, Hongkou, Yangpu; and the suburban districts are Minhang, Baoshan, Jiading, Pudong, Jinshan, Songjiang, Qingpu, Fengxian. We did not include socioeconomic variables in the model over concerns that they would be mediators of the relationship between residency or urbanicity and the outcome, but a sensitivity analysis with education included did not significantly change any parameter estimates.

We included one question to measure each HBM construct, which were measured on a 5-point Likert scale. Perceived prevalence of the disease from the question “How common is [disease type] in your community?”. We measured perceived prevalence instead of the typical construct of perceived susceptibility because of feedback from the qualitative interviews. Previous studies have also made this substitution [29, 30], and have found strong correlations between these two concepts [31, 32].

The vaccine-related questions were asked twice, once for the measles vaccine and once for the pneumococcus vaccine (hereafter indicated as [measles / pneumococcus]). Perceived effectiveness of vaccine from the question, “How effective do you think the [measles/pneumococcus] vaccine is in preventing all cases of [disease type]?”; and perceived safety of the vaccine from the question, “How safe is the [measles/pneumococcus] vaccine?”. Perceived effectiveness of vaccine and perceived safety of vaccine represent the HBM constructs of perceived benefits and barriers to a health-related action, respectively.

We also included questions on disease experience and descriptive norm of vaccination, which are not HBM constructs but which were identified as important in the qualitative research project [28]. Experience with the disease was a binary variable, with the “yes” option being a positive response to any of the following questions: “Have you ever personally contracted [disease type]?”; “Has your child ever contracted [disease type]?”; and “Has any close family member of friend of yours ever contracted [disease type]?”. Finally, perceived norm of vaccination was derived from the question, “Among your social group, how many children do you think are vaccinated against [measles/pneumococcus]?”.

### Statistical analysis

For a descriptive analysis, we used the non-parametric Kruskal-Wallis one-way analysis of variance to test for a significant difference in means for the Likert scale variables across the three disease types (degrees of freedom (df) =2). A Chi-Square test of independence, with the Rao-Scott adjustment to account for the survey design, compared proportions for categorical variables (df = 2, except for caregiver relation, which had df = 4).

For pneumococcal vaccine uptake, two logistic regression models with survey adjustments were run—one for pneumonia-specific perceptions and the other for meningitis-specific perceptions.

To compare how perceptions about measles, pneumonia, and meningitis were differently associated with the outcome vaccine necessity, we created a long-form dataset wherein each individual had 3 observations, one for their perception of each of the three diseases assessed. To account for possible dependence due to each individual yielding three separate observations, we used a generalized estimating equation (GEE) with a binomial distribution and logit link and specified an unstructured within-subject correlation. An interaction term of each predictor variable and a dummy variable for the disease type corresponding to that particular observation was also entered into this model. Significance of the interaction across the 3 disease types was assessed by a Wald chi-square test (df = 2, except for caregiver relation, which had df = 4). Significance was assessed at an α level of 0.05 for all tests, and the precision of odds ratios (OR) was evaluated with 95% confidence intervals (CI). All analyses were weighted based on participants’ probability of selection with respect to urbanicity and residency, and we used SAS version 9.3 (SAS Institute Inc., Cary, North Carolina).

## Results

Out of 734 caregivers approached, 619 caregivers (84.3%) of children who were between 8 months and 7 years of age participated in the survey; nearly two-thirds (64.5%) were mothers of the child, one-quarter (27.6%) were fathers; and 7.8% were other family members, mostly grandmothers. Slightly more than half of the children (51.3%) were male; and 31.3% resided in Shanghai’s urban districts (Table 1). Approximately one-quarter (25.2%) of children had received a pneumococcal vaccine, and nearly all (98.8%) had been administered a measles vaccine.

**Table 1:** Demographic characteristics of 619 children and their caregivers from Shanghai, 2014

Characteristic	Category	Unweighted Count	Weighted proportion (95% CI)
Caregiver relation	Mother	405	64.5 (59.8, 69.3)
	Father	156	27.6 (23.1, 32.2)
	Other	57	7.8 (5.5, 10.1)
Parent’s age^a^	<28 years	105	23.1 (18.3, 27.9)
	28 to <31 years	142	28.0 (23.3, 32.8)
	31 to <34 years	129	24.5 (19.9, 29.1)
	≥35 years	144	24.3 (19.9, 28.8)
Caregiver’s education	≤Middle school	142	24.2 (19.9, 28.6)
	≤High school	93	17.8 (13.8, 21.8)
	Some college	153	23.3 (19.4, 27.3)
	College graduate	227	34.6 (30.1, 39.2)
Family monthly income	<4000 RMB	109	19.1 (15.1, 23.1)
	4000 to <6000 RMB	142	23.9 (19.6, 28.2)
	6000 to <10,000 RMB	156	26.8 (22.4, 31.2)
	≥10,000 RMB	208	30.2 (25.9, 34.5)
Child’s sex	Male	324	51.3 (46.4, 56.2)
	Female	292	48.7 (43.8, 53.6)
Child’s residency	Local	315	43.2 (38.5, 47.8)
	Non-local	303	56.8 (52.2, 61.5)
Township urbanicity	Urban	203	31.3 (26.9, 35.7)
	Suburban	415	68.7 (64.3, 73.1)
Pneumococcal vaccination	Yes	170	25.2 (21.0, 29.3)
	No	448	74.8 (70.7, 79.0)
Measles vaccination	Yes	609	98.8 (98.0, 99.7)
	No	9	1.2 (0.3, 2.0)

*CI* confidence interval

^a^Only for mothers and fathers

All caregiver perception and experience variables were significantly different across the three diseases (Table 2). Most caregivers judged measles (93.2%) and meningitis (92.4%) serious enough to warrant a vaccine, whereas 80.8% thought pneumonia warranted a vaccine. More caregivers (43.3%) had personal experience with pneumonia, compared with 18.6% for measles and only 7.1% for meningitis. Caregivers believed that meningitis was more severe (mean 4.35) than measles (4.07) or pneumonia (4.11); and the perceived prevalence of disease was higher for pneumonia (3.15) than measles (2.30) or meningitis (2.28).

**Table 2:** Perceptions of measles, pneumonia, and meningitis disease and vaccination among caregivers in Shanghai, 2014

	Measles	Pneumonia	Meningitis	*P*-value*
	Mean (SE)	Mean (SE)	Mean (SE)	
Vaccine necessity (%)	93.2 (1.15)	80.8 (1.89)	92.4 (1.34)	<0.0001
Perceived prevalence	2.30 (0.043)	3.15 (0.047)	2.28 (0.037)	<0.0001
Disease experience (%)	18.6 (1.96)	43.3 (2.44)	7.1 (1.40)	<0.0001
Perceived norm	4.03 (0.045)	3.35 (0.051)	3.35 (0.051)	<0.0001
Perceived effectiveness	3.81 (0.033)	3.58 (0.032)	3.56 (0.035)	<0.0001
Perceived safety	3.92 (0.036)	3.81 (0.034)	3.81 (0.034)	0.0404

*SE* standard error

*For Likert scale variables, the *P*-value is the Kruskal-Wallis test. For dichotomous variables, the *P*-value is from the Rao-Scott Chi-Square Test

Perceived necessity of a pneumonia vaccine was the strongest predictor of pneumococcal vaccine uptake in the model with pneumonia-specific perceptions (OR: 2.67, 95% CI: 1.27, 5.63) (Table 3). Perceived safety of vaccination was a significant predictor in the models for both pneumonia-specific (OR: 2.39, 95% CI: 1.57, 3.63) and meningitis-specific perceptions (OR: 2.12, 95% CI: 1.24, 3.63).

**Table 3:** Pneumonia- or meningitis-specific perceptions and pneumococcal vaccine uptake among 602 caregivers in Shanghai, 2014

	Pneumonia	Meningitis
	OR (95% CI)	OR (95% CI)
Perceived vaccine necessity	2.67 (1.27, 5.63)	1.45 (0.52, 3.99)
Perceived prevalence	1.10 (0.84, 1.44)	0.97 (0.73, 1.31)
Disease experience		
Yes vs No	1.16 (0.70, 1.94)	1.32 (0.55, 3.17)
Perceived norm	1.16 (0.92, 1.46)	1.21 (0.96, 1.53)
Perceived effectiveness	0.91 (0.61, 1.35)	0.95 (0.61, 1.47)
Perceived safety	2.39 (1.57, 3.63)	2.12 (1.24, 3.63)
Residency		
Non-local vs local	1.01 (0.63, 1.60)	0.97 (0.62, 1.52)
Urbanicity		
Outer vs inner district	1.10 (0.69, 1.78)	1.31 (0.82, 2.08)
Caregiver relation		
Father vs mother	0.71 (0.41, 1.24)	0.67 (0.39, 1.17)
Other vs mother	1.54 (0.71, 3.32)	1.20 (0.56, 2.53)

*OR* odds ratio, *CI* confidence interval

Results from the multivariable model of vaccine necessity are shown in Table 4. An increase in perceived norm of vaccination was associated with 1.97 times greater odds of measles vaccine necessity (95% CI: 1.50, 2.59) and 1.53 times greater odds of pneumonia vaccine necessity (95% CI: 1.23, 1.91). Perceived safety of vaccination was positively associated with measles (OR: 2.35; 95% CI: 1.26, 4.38), pneumonia (OR: 1.62; 95% CI: 1.04, 2.52), and meningitis vaccine necessity (OR: 2.11, 95% CI: 1.31, 3.40). Perceived prevalence of disease was not associated with necessity for vaccination against measles, pneumonia, or meningitis.

**Table 4:** Predictors of vaccine necessity for three diseases among 602 caregivers in Shanghai, 2014

	Measles	Pneumonia	Meningitis	P-value^a^
	OR (95% CI)	OR (95% CI)	OR (95% CI)	
Perceived prevalence	1.25 (0.85, 1.83)	1.18 (0.91, 1.53)	1.08 (0.65, 1.80)	0.9022
Disease experience				0.2576
Yes vs No	1.48 (0.58, 3.78)	0.76 (0.46, 1.26)	0.34 (0.10, 1.10)	
Perceived norm	1.97 (1.50, 2.59)	1.53 (1.23, 1.91)	1.13 (0.80, 1.61)	0.0753
Perceived effectiveness	1.31 (0.69, 2.49)	4.05 (2.61, 6.31)	1.17 (0.57, 2.42)	0.0088
Perceived safety	2.35 (1.26, 4.38)	1.62 (1.04, 2.52)	2.11 (1.31, 3.40)	0.5349
Residency				0.4511
Non-local vs local	1.77 (0.84, 3.73)	1.70 (1.01, 2.88)	1.06 (0.48, 2.36)	
Urbanicity				0.0016
Suburban vs urban	0.37 (0.15, 0.92)	1.74 (1.01, 3.00)	0.79 (0.32, 1.95)	
Caregiver relation				0.1886
Father vs mother	0.38 (0.17, 0.84)	1.21 (0.64, 2.27)	1.07 (0.44, 2.56)	
Other vs mother	0.20 (0.06, 0.65)	0.50 (0.21, 1.17)	0.45 (0.14, 1.48)	

*OR* odds ratio, *CI* confidence interval

^a^Wald chi-square test for overall interaction (df = 2, except for caregiver relation, which had df = 4). Results in this table are from a single, multivariable logistic regression model

The strength of the associations between most explanatory variables and vaccine necessity did not vary significantly by disease. However, the relationship between perceived effectiveness of vaccination and necessity of vaccination did vary by disease (*P* = 0.0088); for pneumonia this was a positive association (OR: 4.05; 95% CI: 2.61, 6.31), whereas for measles and meningitis there was no association. There was also a significant interaction between urbanicity and disease (*P* = 0.0016); people living in suburban districts were more likely to consider the pneumonia vaccine necessary (OR: 1.74; 95% CI: 1.01, 3.00), whereas the opposite relationship (OR: 0.37; 95% CI: 0.15, 0.92) was found for the measles vaccine. Additionally, although the interaction term for residency was not significant, non-locals had higher odds of pneumonia vaccine necessity than locals (OR: 1.70; 95% CI: 1.01, 2.88), whereas there was not a significant association between residency and either measles (OR: 1.77; 95% CI: 0.84, 3.73) or meningitis vaccine necessity (OR: 1.06; 95% CI: 0.48, 2.36).

## Discussion

In order to increase coverage of newer, pediatric vaccines in middle income countries, it is necessary to develop a better understanding of the relationships between caregiver perceptions of a disease and its vaccine. In this cross-sectional survey of parents and grandparents in Shanghai, only a minority of children had been administered a pneumococcal vaccine, even though most of their caregivers believed that pediatric pneumonia and meningitis vaccines were necessary. Moreover, whereas the vast majority of caregivers thought measles and meningitis were serious enough to warrant a vaccine, a lesser amount held similar beliefs for pneumonia. Previous studies have also shown that parents generally do not consider pneumonia vaccines as important as other vaccines. In the Netherlands, Hak et al. found that fewer parents had a positive attitude towards pneumonia vaccines than other vaccines, such as those for hepatitis B or tuberculosis [33]. Bedford and Lansley similarly reported that fewer British parents would accept a pneumococcal vaccine than a meningococcal vaccine. They postulated that this difference in acceptance came from parents associating meningococcus with meningitis and pneumococcus with pneumonia, and subsequently believing that meningitis was more clinically severe than pneumonia [34], which is corroborated by our study in comparing perceptions of pneumococcal meningitis and pneumococcal pneumonia.

In this study, perceived vaccine necessity but not perceived prevalence of pneumonia, was positively associated with pneumococcal vaccine uptake. Both our study and a study on pediatric dysentery vaccination by Chen et al. did not observe a relationship between perceived prevalence and vaccine need [18], suggesting that, for Chinese parents, perceptions about a disease’s threat primarily derive from concerns about severity and is not necessarily based on their understanding of how common the disease is within the community. It is possible that Chinese caregivers may view the threat of diseases in a fundamentally different way compared to caregivers in other countries because the one-child policy likely results in heightened focus from parents on one child [35]. Chinese caregivers’ high investment in their child’s safety may explain why pneumonia vaccine necessity, a measure of disease severity, was a strong predictor of pneumococcal vaccine uptake and why perceived vaccine safety was strongly and positively associated with all vaccine outcomes that we considered.

Besides perceived safety of vaccination, necessity of pneumonia vaccination was also associated with pneumococcal vaccine uptake. Vaccine necessity could be an important mediator in the pathway between disease perceptions and vaccine uptake, and we may observe stronger associations between disease perceptions and vaccine necessity than disease perceptions and vaccine uptake because vaccine necessity is more proximal to these perceptions. The lack of significant associations between perceptions of meningitis and pneumococcal vaccination could result from caregivers not being aware that pneumococcal vaccines can protect against some forms of meningitis.

Most of the HBM constructs and other beliefs under consideration had a similar relationship with vaccine necessity, regardless of disease. Because we saw consistent associations between HBM constructs and vaccine necessity, we conclude there was a common mechanism underlying how Chinese parents decided which vaccines are necessary, in the context of their perceptions about the disease and the vaccine. However, the strength of the relationship between perceived effectiveness of vaccination and vaccine necessity did differ by disease. For measles, perceived effectiveness of vaccination was not an important determinant of vaccine necessity, perhaps because measles vaccine is mandatory. In contrast, because the pneumococcal vaccine requires payment from caregivers, they may only feel their children need it if the vaccine is effective. We may not see any association for meningitis because the pneumococcal vaccine in China is marketed as a pneumonia vaccine, not a meningitis vaccine, and we conjecture that caregivers have little understanding of how the pneumococcal vaccine can prevent some forms of meningitis.

Given the high uptake of EPI vaccines in China, adding pneumococcal vaccination to the EPI schedule will undoubtedly increase coverage, however, we have no indication when or if this will happen, especially since PCV7 was taken off the market in China in 2015 [36]. Measles vaccine is an EPI vaccine but pneumococcal vaccine is not, and this difference prevented us from considering other factors that influence vaccine uptake in other countries. First, measles vaccine is free in Shanghai but pneumococcal vaccine requires payment. This could significantly impact decisions; in a 7-country survey of parents, support for a vaccine decreased by 14% if the vaccine required payment [37]. Second, China has focused tremendous efforts on measles elimination, and hundreds of millions of children have been vaccinated against measles during supplementary immunization activities within the past decade [38]. There has not been a comparable effort for pneumococcal vaccination. Therefore, the Chinese public is receiving more information about measles than about pneumonia or meningitis.

Non-locals and suburban dwellers have a number of different experiences and attributes which distinguish them from their local or urban counterparts. They may have different experiences with disease, given disparities in treatment or ability to interface with health care providers. Notably, quality and density of health care diminishes outside of urban areas in cities [16], and non-locals access health care services much less than locals [15]. Previous studies have shown that non-locals have lower vaccination coverage than locals, and suburban children have worse vaccination outcomes than those in urban districts, for both EPI and non-EPI vaccines [7, 39, 40]. Yet we found that non-locals and suburban dwellers had greater odds of considering pneumonia vaccines as necessary compared to locals and urban dwellers. That these subpopulations think that pneumonia vaccines are necessary but do not receive them could result from the cost, and financial incentives from the government may be necessary to increase vaccination coverage, particularly in these poorer populations where there is a demand for vaccination.

This study provides a framework for developing a better understanding of the context driving demand for a vaccine. As Nichter notes, there is a difference between passive acceptance of and active demand for vaccinations [41]. With passive acceptance, the populace attains high vaccination coverage only after the public health sector devotes intensive resources towards promoting a certain vaccine. By contrast, in the latter, a well-informed public perceives the need for vaccination and drives demand for immunization services. In our survey, perceived necessity of vaccination by caregivers was higher for measles and meningitis than it was for pneumonia. This implies that even if pneumonia vaccination were added to the EPI schedule, active demand could be lower than for other vaccines. Thus, uptake would be driven by pressure from the public health sector and not from caregivers demanding the vaccine.

### Strengths and limitations

The study has both important strengths and limitations. One strength was the purposeful sampling of people by residency to account for an important demographic group in Shanghai. However, within each township’s immunization clinic, we selected a convenience sample. This means that the study population is biased towards a population with more positive views towards immunization services. Additionally, we only used one item to measure each HBM construct, and therefore could not minimize measurement error by formulating latent constructs.

This study evaluated perceptions of meningitis and pneumococcal vaccinations, but a Hib vaccine and meningococcal vaccine are also available in China to protect against these diseases. The etiology of pneumonia and meningitis in China is poorly understood [5, 42, 43], but it is likely that Hib results in comparable rates of pneumonia morbidity and mortality as pneumococcus [5, 44], and that the cause of meningitis morbidity in China is somewhat equally divided between meningococcus and pneumococcus [5, 45]. A caregiver’s perception of vaccine necessity could therefore be colored by the other vaccines already on the market, and we hypothesize that caregivers would be less apt to consider a vaccine necessary if they also thought that that disease could be caused by a number of different infections.

## Conclusions

Given the enormous toll of pneumococcal disease in China [3, 5], widespread pneumococcal vaccination could improve child health and save lives. China has spent tremendous resources on measles elimination [38], but measles elimination efforts should could be combined with other immunization initiatives [46], such as educating caregivers about the benefits of other vaccines. In particular, because more people thought that a meningitis vaccine was necessary than a pneumonia vaccine, promotional materials for pneumococcal vaccines could focus disease severity and on meningitis, the more severe clinical presentation of pneumococcal disease.

Future studies could take a longitudinal look at attitudes towards a disease, the desire to obtain a vaccine, and, finally, actual vaccination. Additionally, both the relationship between the patient and the provider and how the provider approaches talking about vaccination are important [47], warranting further research on health care workers in China. As more vaccines are introduced into the EPI schedule in China, providers will be an important conduit of information about the risk of disease and the safety and effectiveness of vaccination.

## Additional files

Additional file 1: English version of questionnaire, April 3, 2014. (PDF 242 kb)

## Abbreviations

CI: confidence interval
df: degrees of freedom
DTP: diphtheria-tetanus-pertussis vaccine
EPI: Expanded Program on Immunization
GEE: Generalized Estimating Equations
HBM: Health Belief Model
Hib: *Haemophilus influenzae* type b
OR: Odds ratio
PCV7: 7-valent pneumococcal conjugate vaccine
PPS: Probability proportionate to size
PPSV23: 23-valent pneumococcal polysaccharide vaccine
RMB: Renminbi
SE: standard error
