# Supplementary material for: Perceptions of measles, pneumonia, and meningitis vaccines among caregivers in Shanghai, China, and the health belief model: a cross-sectional study
Source: BMC Pediatr. 2017 Jun 12;17:143. doi: 10.1186/s12887-017-0900-2 (PMC5468991; doi:10.1186/s12887-017-0900-2)
Supplement: Supplementary file 1 — English version of questionnaire, April 3, 2014. (PDF 242 kb) [file 12887_2017_900_MOESM1_ESM.pdf]

Additional files

English version of questionnaire, April 3, 2014

Individual questionnaire

You are invited to participate in a survey of parents. This survey is sponsored by the Shanghai Centers for Disease Control and Prevention and the University of Michigan in the United States. In total, over 600 parents of young children will be selected from across Shanghai. This survey will research parents' attitudes towards vaccination, particularly vaccines requiring payment. This information will help guide interventions for increasing vaccination coverage. Your response will be anonymous and your decision to participate in this survey or not will not affect your treatment at this clinic. This survey should take 20 minutes, and you will receive 30 renminbi for completing every question on this survey.

We first want to ask about the child you brought to the clinic today.

1. What is your relationship to the child?
   - 1☐ Mother
   - 2☐ Father
   - 3☐ Other (Please describe ______________________)

2. What is the birthdate of the child you brought to the clinic today?

   ___ ___ ___ ___ - ___ ___ - ___ ___
   Y Y Y Y M M D D

3. What is the sex of the child you brought to the clinic today?
   - 1☐ Male
   - 2☐ Female

Next we want to ask you some questions about the immunization clinic and about vaccinations that require payments.

4. How long have you known the doctors at your immunization clinic?

   ____ years ____ months

5. When deciding on whether to get a vaccine you must pay for, how trustworthy are recommendations from your doctors at the immunization clinic?

Not at all trustworthy				Extremely trustworthy
1☐	2☐	3☐	4☐	5☐

6. When deciding on whether to get a vaccine you must pay for, how important is it for you to look online for information about vaccines yourself?

Not at all important				Extremely important
1☐	2☐	3☐	4☐	5☐

7. When deciding on whether to get a vaccine you must pay for, how important is it for you to consult family and friends?

Not at all important				Extremely important
1☐	2☐	3☐	4☐	5☐

8. When deciding on whether to get a vaccine you must pay for, how important is it for you to consult parents in your social group?

Not at all important				Extremely important
1☐	2☐	3☐	4☐	5☐

9. How worried are you with giving your child two or more vaccines at the same time?

Not at all worried				Extremely worried
1☐	2☐	3☐	4☐	5☐

10. How worried are you with giving your child vaccines when the child is under 6 months?

Not at all worried				Extremely worried
1☐	2☐	3☐	4☐	5☐

11. How important is it for you to give your child vaccines on time?

Not at all important				Extremely important
1☐	2☐	3☐	4☐	5☐

12. What is the largest number of vaccine shots that you are willing to give your child during the same clinic visit?

    0☐ None
    1☐ 1
    2☐ 2
    3☐ 3
    4☐ More than 3
    5☐ As many as recommended by the doctor

13. How often are you willing to come to the immunization clinic to get your child a vaccine if recommended to do so?
    - 0☐ As often as needed
    - 1☐ Once a week
    - 2☐ A couple of times each month
    - 3☐ Once a month
    - 4☐ Every other month
    - 5☐ A few times a year at most

14. How important is it for you to get **every** vaccine recommended by your doctor?

Not at all important				Extremely important
1☐	2☐	3☐	4☐	5☐

15. How trustworthy is **traditional Chinese medicine** to cure infectious disease?

Not at all trustworthy				Extremely trustworthy
1☐	2☐	3☐	4☐	5☐

16. How trustworthy is **Western medicine** to cure infectious disease?

Not at all trustworthy				Extremely trustworthy
1☐	2☐	3☐	4☐	5☐

17. Do you think that the effectiveness of Chinese-made vaccines is different than the effectiveness of foreign-made vaccines?
    1☐ Yes
    2☐ No
    3☐ I do not know

18. How effective are foreign-made vaccines in Chinese children?

Not at all effective				Extremely effective
1☐	2☐	3☐	4☐	5☐

19. How effective are Chinese-made vaccines in Chinese children?

Not at all effective				Extremely effective
1☐	2☐	3☐	4☐	5☐

20. Do you think that the safety of Chinese-made vaccines is different than the safety of foreign-made vaccines?
    1☐ Yes
    2☐ No
    3☐ I do not know

21. How safe are foreign vaccines in Chinese children?

Not at all safe				Extremely safe
1☐	2☐	3☐	4☐	5☐

22. How safe are Chinese-made vaccines in Chinese children?

Not at all safe				Extremely safe
1☐	2☐	3☐	4☐	5☐

23. When deciding on whether to get a vaccine you must pay for, which of these statements best applies to you?
    1☐ My child receives all vaccines that require payment at the earliest time that these vaccines are recommended.
    2☐ My child receives all vaccines that require payment, but I may choose to delay when my child gets at least some of these vaccines.
    3☐ I may choose to refuse one or more of these vaccines that require payment.
    4☐ I refuse all vaccines that require payment.

Next, we are going to ask you about two different diseases and their associated vaccines: measles and pneumococcus.

24. Do you know what measles is?
   1☐ Yes
   2☐ No

**MEASLES**
Measles virus causes rash, cough, runny nose, eye irritation, and fever. It can lead to ear infection, pneumonia, seizures (jerking and staring), brain damage, and death.

25. Have you ever personally contracted measles?
   1☐ Yes
   2☐ No
   3☐ I do not know

26. Has your child ever contracted measles?
   1☐ Yes
   2☐ No
   3☐ I do not know

27. Has any close family member or friend of yours ever contracted measles?
   1☐ Yes
   2☐ No
   3☐ I do not know

28. How **serious** of a disease do you think is measles?

Not at all serious				Extremely serious
1☐	2☐	3☐	4☐	5☐

29. Do you think that measles is a serious enough disease to warrant a vaccine?
   1☐ Yes
   2☐ No

30. How **common** is measles in your community?

Not at all common				Extremely common
1☐	2☐	3☐	4☐	5☐

31. How **effective** do you think the measles vaccine is in preventing all cases of measles?

Not at all effective				Extremely effective
1☐	2☐	3☐	4☐	5☐

32. Among your social group, **how many children** do you think are vaccinated against measles?

None		About half		All
1☐	2☐	3☐	4☐	5☐

33. How **safe** is the measles vaccine?

Not at all safe				Extremely safe
1☐	2☐	3☐	4☐	5☐

34. After getting a measles vaccine, how likely is your child to get **a mild reaction**, like a mild fever?

Not at all likely				Extremely likely
1☐	2☐	3☐	4☐	5☐

35. After getting a measles vaccine, how likely is your child to get **a moderate or major reaction**, like a seizure?

Not at all likely				Extremely likely
1☐	2☐	3☐	4☐	5☐

36. Do you know what pneumonia is?
- 1☐ Yes
- 2☐ No

37. Do you know what meningitis is?
- 1☐ Yes
- 2☐ No

PNEUMOCOCCUS

Pneumococcal disease is caused by infection with *Streptococcus pneumoniae* bacteria. These bacteria can spread from person to person through close contact. Pneumococcal disease can lead to severe health problems, including pneumonia, blood infections, and meningitis. Meningitis is an infection of the covering of the brain. Pneumococcal meningitis is fairly rare, but it leads to other health problems, including deafness and brain damage.

38. Have you ever personally contracted pneumonia?
- 1☐ Yes
- 2☐ No
- 3☐ I do not know

39. Has your child ever contracted pneumonia?
- 1☐ Yes
- 2☐ No
- 3☐ I do not know

40. Has any close family member or friend of yours ever contracted pneumonia?
- 1☐ Yes
- 2☐ No
- 3☐ I do not know

41. How **serious** of a disease do you think is pneumonia?

Not at all serious				Extremely serious
1☐	2☐	3☐	4☐	5☐

42. Do you think that pneumonia is a serious enough disease to warrant a vaccine?
- 1☐ Yes
- 2☐ No

43. How **common** is pneumonia in your community?

Not at all common				Extremely common
1☐	2☐	3☐	4☐	5☐

44. How **effective** do you think the pneumococcus vaccine is in preventing all cases of pneumonia?

Not at all effective	2☐	3☐	4☐	Extremely effective

1☐ 5☐

45. Have you ever personally contracted meningitis?
1☐ Yes
2☐ No
3☐ I do not know

46. Has your child ever contracted meningitis?
1☐ Yes
2☐ No
3☐ I do not know

47. Has any close family member or friend of yours ever contracted meningitis?
1☐ Yes
2☐ No
3☐ I do not know

48. How **serious** of a disease do you think is meningitis?

Not at all serious				Extremely serious
1☐	2☐	3☐	4☐	5☐

49. Do you think that meningitis is a serious enough disease to warrant a vaccine?
1☐ Yes
2☐ No

50. How **common** is meningitis in your community?

Not at all common				Extremely common
1☐	2☐	3☐	4☐	5☐

51. How **effective** do you think the pneumococcus vaccine is in preventing all cases of meningitis?

Not at all effective				Extremely effective
1☐	2☐	3☐	4☐	5☐

52. Among your social group, **how many children** do you think are vaccinated against pneumococcus?

None		About half		All
1☐	2☐	3☐	4☐	5☐

53. How **safe** is the pneumococcus vaccine?

Not at all safe				Extremely safe
1☐	2☐	3☐	4☐	5☐

54. After getting a pneumococcus vaccine, how likely is your child to get **a mild reaction**, like a mild fever?

Not at all likely				Extremely likely
1☐	2☐	3☐	4☐	5☐

55. After getting a pneumococcus vaccine, how likely is your child to get **a moderate or major reaction**, like a seizure?

Not at all likely				Extremely likely
1☐	2☐	3☐	4☐	5☐

56. How much are you willing to pay for the 7-valent pneumococcus vaccine (called PCV7)?
   - 0☐ 0 yuan
   - 1☐ 50 yuan
   - 2☐ 100 yuan
   - 3☐ 200 yuan
   - 4☐ 500 yuan
   - 5☐ 700 yuan
   - 6☐ Over 700 yuan

The last set of questions are about you.

57. How often do you work each week?
   - 1☐ I currently do not work
   - 2☐ 1 day
   - 3☐ 2 days
   - 4☐ 3 days
   - 5☐ 4 days
   - 6☐ 5 days
   - 7☐ More than 5 days

58. What is your family's monthly income?
   - 1☐ <2, 000 元
   - 2☐ 2, 000 to 3, 999 元
   - 3☐ 4, 000 to 5, 999 元
   - 4☐ 6, 000 to 9, 999 元
   - 5☐ 10, 000 to 19, 999 元
   - 6☐ Over 20, 000 元

59. What is your highest education level?
   - 1☐ None
   - 2☐ Elementary
   - 3☐ Middle school
   - 4☐ High school
   - 5☐ Some college
   - 6☐ College or above

60. What is your residency?
   - 1☐ Same city and same district
   - 2☐ Same city, different district
   - 3☐ Different city

61. How long have you lived in this city?
   - 0☐ My entire life

   ____ years ____ months

62. How long do you plan on staying in this city?
   - 0☐ Indefinitely

   ____ years ____ months

63. What is your home city?
- 0☐ Shanghai
- 1☐ Somewhere other than Shanghai:

  _____________ Province

If your home city is Shanghai, you do not need to answer the following question, and you have finished the survey. Please answer the following question if your home city is not Shanghai.

64. How often do you return to your home city?
- 1☐ More than once a year
- 2☐ Once a year
- 3☐ Once every couple years
- 4☐ Fewer than once every couple years
- 5☐ Never

Thank you very much for your participation! Please return this form to a staff member. Have your vaccination booklet available and they will record some information about vaccinations that your child has received.

The following information should be filled out by staff members looking at the child's vaccination booklet. Please give the questionnaire and your child's vaccination booklet to the staff members, and wait while they fill out these last questions.

65. What measles vaccinations (including MM, MR, or MMR) has the child received?

Type of vaccine	Date of vaccination
1☐ Measles<br>2☐ MR<br>3☐ MMR<br>4☐ MM	___ ___ ___ ___ - ___ ___ - ___ ___<br>Y Y Y Y M M D D
1☐ Measles<br>2☐ MR<br>3☐ MMR<br>4☐ MM	___ ___ ___ ___ - ___ ___ - ___ ___<br>Y Y Y Y M M D D
1☐ Measles<br>2☐ MR<br>3☐ MMR<br>4☐ MM	___ ___ ___ ___ - ___ ___ - ___ ___<br>Y Y Y Y M M D D
1☐ Measles<br>2☐ MR<br>3☐ MMR<br>4☐ MM	___ ___ ___ ___ - ___ ___ - ___ ___<br>Y Y Y Y M M D D
1☐ Measles<br>2☐ MR<br>3☐ MMR<br>4☐ MM	___ ___ ___ ___ - ___ ___ - ___ ___<br>Y Y Y Y M M D D

66. What pneumococcal vaccinations (including PCV7 and PPSV23) has the child received?

Type of vaccine	Date of vaccination
1☐ PCV7<br>2☐ PPSV23	___ ___ ___ ___ - ___ ___ - ___ ___
1☐ PCV7<br>2☐ PPSV23	___ ___ ___ ___ - ___ ___ - ___ ___
1☐ PCV7<br>2☐ PPSV23	___ ___ ___ ___ - ___ ___ - ___ ___
1☐ PCV7<br>2☐ PPSV23	___ ___ ___ ___ - ___ ___ - ___ ___
